# A model combining age, equivalent uniform dose and IL-8 may predict radiation esophagitis in patients with non-small cell lung cancer

**DOI:** 10.1016/j.radonc.2017.12.026

**Published:** 2018-03

**Authors:** Shulian Wang, Jeff Campbell, Matthew H. Stenmark, Paul Stanton, Jing Zhao, Martha M. Matuszak, Randall K. Ten Haken, Feng-Ming Kong

**Affiliations:** a State Key Laboratory of Molecular Oncology, Department of Radiation Oncology, National Cancer Center/Cancer Hospital, Chinese Academy of Medical Sciences and Peking Union Medical College, Beijing, China; b Department of Radiation Oncology, GRU Cancer Center and Medical College of Georgia, Augusta, GA, United States; c Department of Radiation Oncology, University of Michigan; d Department of Radiation Oncology, Indiana University, United States

**Keywords:** Lung neoplasm, Non-small cell, Radiation esophagitis, Cytokines

## Abstract

**Background and purpose:**

To study whether cytokine markers may improve predictive accuracy of radiation esophagitis (RE) in non-small cell lung cancer (NSCLC) patients.

**Materials and methods:**

A total of 129 patients with stage I-III NSCLC treated with radiotherapy (RT) from prospective studies were included. Thirty inflammatory cytokines were measured in platelet-poor plasma samples. Logistic regression was performed to evaluate the risk factors of RE. Stepwise Akaike information criterion (AIC) and likelihood ratio test were used to assess model predictions.

**Results:**

Forty-nine of 129 patients (38.0%) developed grade ≥2 RE. Univariate analysis showed that age, stage, concurrent chemotherapy, and eight dosimetric parameters were significantly associated with grade ≥2 RE (*p* < 0.05). IL-4, IL-5, IL-8, IL-13, IL-15, IL-1α, TGFα and eotaxin were also associated with grade ≥2 RE (*p* <0.1). Age, esophagus generalized equivalent uniform dose (EUD), and baseline IL-8 were independently associated grade ≥2 RE. The combination of these three factors had significantly higher predictive power than any single factor alone. Addition of IL-8 to toxicity model significantly improves RE predictive accuracy (*p* = 0.019).

**Conclusions:**

Combining baseline level of IL-8, age and esophagus EUD may predict RE more accurately. Refinement of this model with larger sample sizes and validation from multicenter database are warranted.

Radiation esophagitis (RE) is a common acute toxicity for non-small cell lung cancer (NSCLC) patients treated with radiation therapy (RT). It occurs during RT and often persists for several weeks after completion of RT. Grade ≥2 RE developed in about 50% of patients [1], which is clinically significant and may compromise the treatment result due to medical intervention or sometimes a treatment break. Identification of predictive factors for RE facilitates safe delivery of optimal prescribed dose to an individual patient. A meta-analysis published in 2013 [1] showed that V60 of esophagus provides the best predictive ability for RE among all clinical and dosimetric factors, although clinical stage, nodal stage, performance status, type of chemotherapy, RT schedule (total number of fractions, dose per fraction) are statistically predictive of RE.

Risk of RE in each individual patient is often not explained by these clinical factors. In clinical practice, patients with similar clinical factors and dose volume distributions to the esophagus commonly have different risks of RE. Additional biomarkers reflecting inherent radiosensitivity of esophagus might improve the predictive potential, including single nucleotide polymorphisms (SNPs) in the TGFβ1 gene [2–4], certain serum miRNA [5], or certain genetic parameters [6]. Currently, no reliable biomarker has been found that can be used in clinic as early predictors of RE.

Our hypothesis is (1) baseline levels of inflammatory cytokines, or their dynamic changes during RT, correlate with the risk of RE; and (2) the addition of inflammatory cytokines to other dosimetric and clinical factors improves the prediction of RE.

## Methods

The study population included 129 patients with newly diagnosed stage I–III NSCLC, consecutively enrolled in 3 prospective Institutional Review Board-approved NSCLC studies conducted at the University of Michigan Cancer Center and the Veterans Affairs Medical Center, Ann Arbor, MI: (1) a phase 1/2 study of RT dose escalation (limited to a lung normal tissue complication probability [NTCP] value of <15%) with concurrent chemotherapy and 2 consecutive studies using (2) functional imaging and (3) biomarkers to assess outcome (ClinicalTrials.gov: NCT01190527, NCT00603057).

All patients received definitive radiotherapy. Radiotherapy was delivered using three-dimensional conformal technique. A median total dose of 70 Gy (range, 44–85.5 Gy) was administered in 2.0–2.9 Gy daily fractions over 4–7 weeks using 6 or 6/16 MV photons. The prescribed dose covered 95% planning target volume (PTV). In patients treated under dose escalated protocols, the effective volume of esophagus irradiated (Veff) computed with a normalization dose biologically equivalent to 72 Gy in 2 Gy fractions was limited to less than 1/3 of the esophagus.

On planning CT, the esophagus was defined to include the esophageal wall and lumen which was contoured from cricoid to gastroesophageal junction for dosimetric computation. Dose–volume histograms (DVH) for the esophagus were then calculated after conversion of doses to their 2 Gy equivalents (EQD2) using the linear quadratic model with alpha/beta of 10. The following dosimetric parameters of esophagus were retrieved or converted from the DVH: The total volume of esophagus (Vtotal); the maximum dose (Dmax); the mean dose (Dmean); the relative volume of esophagus receiving >60 Gy (rV60); the absolute volume of esophagus which received >60 Gy (aV60), and >70 Gy (aV70); generalized equivalent uniform dose (EUD) and NTCP. Lyman model parameters (TD50 = 68 Gy, *n* = 1/a = 0.06, *m* = 0.11) were used to compute EUD and NTCP [7].

All patients were evaluated prospectively weekly during RT, with follow-up evaluations at 1 month after completion of RT, every 3 months for 1 year, every 6 months at second year and yearly afterward. At each follow-up, patients underwent a history review and physical examination as well as a chest CT scan. Treatment-related toxicity including RE was evaluated and graded at the time of visit by the treating physician according to Common Terminology Criteria for Adverse Events version 3.0. The maximum esophagitis grade was recorded for each patient. The incidence of RE was calculated as the number of patients with RE divided by the total number of patients. The primary endpoint in this study was grade ≥2 RE, including dysphagia and odynophagia.

Serial blood samples were collected with K2EDTA (dikalium salt of ethylenediaminetetraacetic acid) anticoagulant within two weeks prior to RT (pre) and at 2 weeks (2w) and 4 weeks (4w) during RT. Blood samples were placed in ice immediately after collection, centrifuged within 6 h of collection at 3000 g for 30 min (4°C), and supernatants were collected and stored at −80°C until use.

Thirty cytokines related to inflammatory process and with available commercial kits were selected for this study. Measurements of cytokines were performed in these platelet-poor plasma samples. Commercial Human Cytokine/Chemokine Magnetic Bead Panel kits (MILLIPLEX^®^ MAP, Cat. # HCYTMAG-60K-PX29 (pre-mixed); Millipore, Billerica, MA) were used to measure the levels of 29 cytokines, including EGF, Eotaxin, Fractalkine, G-CSF, GM-CSF, IFNγ, IL-10, IL-12P40, IL-12P70, IL-13, IL-15, IL-17, IL-1RA, IL-1α, IL-1β, IL-2, IL-4, IL-5, IL-6, IL-7, IL-8, IP-10, MCP-1, MIP-1α, MIP-1β, sCD40L, TGF-α, TNFα, VEGF. TGF-β1 level was measured by molecule-specific enzyme-linked immunosorbent assay (Human TGFβ1 DuoSet kit, R&D Systems Inc., Minneapolis, MN). All sample tests were run in duplicate, according to the manufacturer’s instructions.

Univariate logistic regression was performed with Grade 2 or more RE as a dependent variable to determine potential predictors of RE, including clinical factors, dosimetric parameters, and cytokine levels (pre, 2w, 4w) and ratios at 2 and 4 weeks to baseline (2w/pre and 4w/pre). All cytokine levels were log-transformed for use in the logistic regression models because of their skewed distribution. All significant factors were considered for a multivariate model based upon their significance in univariate regression. Factors were selected using a forward stepwise selection procedure based upon the Akaike information criterion (AIC) as well as the likelihood ratio test (LRT). Because of the high degree of correlation between dosimetric measures, one dosimetric variable was selected by evaluating both the *p*-value and AIC for univariate models with dosimetric factors. The combined model was also compared with univariate models comparing AUC of each model’s Receiver operating characteristic (ROC) curve from the data. Cutoff points were created for age, EUD, and baseline cytokine like IL-8 based on maximum values of the sum of sensitivity and specificity. All *p*-values were from two-sided test.

## Results

Table 1 shows the baseline clinical characteristics. The median age of the 129 patients was 66 years (range, 40–92 years) with ECOG performance status 0–2. All patients received definitive RT with or without concurrent chemotherapy. Carboplatin and paclitaxel were used as concurrent chemotherapy regimen in 88% of patients.

Forty out of 129 patients (31.0%) developed grade 1 RE, 33 patients (25.6%) grade 2, 15 patients (11.6%) grade 3, and one patient (0.8%) grade 4. There were no esophagitis-related deaths. Altogether, 49 patients (38.0%) had grade ≥2 RE. The median time from the start of RT to the development of grade ≥2 RE was 4.0 weeks (range; 1–20 weeks). It was 3.0 weeks (range; 1–18 weeks) for grade 2 RE and 4.5 weeks (range; 2–20 weeks) for grade 3–4 RE.

Age, gender, smoking history, stage, RT dose and concurrent chemotherapy were evaluated for the association with RE, with age and RT dose as continuous variables. Younger age (OR: 0.94, 95% CI: 0.91–0.98; *p* = 0.003), stage III disease (OR: 4.70, 95% CI: 1.00–21.70; *p* = 0.048), use of concurrent chemotherapy (OR: 4.13, 95% CI: 1.45–11.70; *p* = 0.010) were significantly associated with higher risk of grade ≥2 RE.

One hundred and three patients had dosimetric parameters of esophagus available. Higher NTCP, Dmax, Dmean, EUD, rV60, aV60, aV70 were associated with higher risk of grade ≥2 RE (Table 2). These dosimetric parameters were all significantly correlated with each other (*p* < 0.001).

**Fig. 1:**
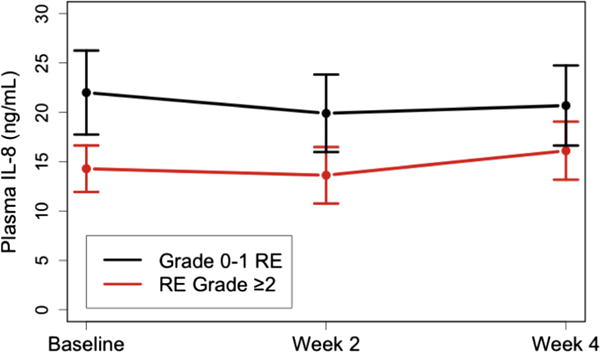
IL-8 levels for patients with and without Grade ≥2 RE. RE = radiation esophagitis; RT = radiotherapy.

**Fig. 2:**
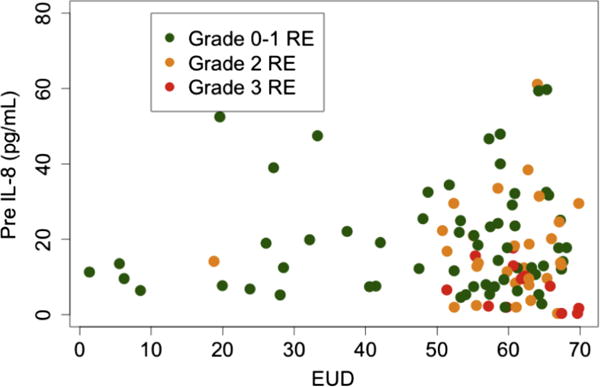
Scatted plots of values of pre-IL-8 and EUD of each patient with and without RE. RE = radiation esophagitis; EUD = equivalent uniform dose.

TGF-β1 was measured before RT in 129, at 2w in 128, and at 4w in 107 patients. The other 29 cytokines were measured before RT and 2w in 129, at 4w in 127 patients. The association of the levels of 30 cytokines with grade ≥2 RE is shown in Table 3. IL-4, IL-5, IL-8, IL-13, IL-15, IL-1α, TGFα, TGFβ1 and eotaxin at different time points were associated with grade ≥2 RE (*p* < 0.1). Under univariate analysis, baseline levels of IL-8, IL-13 and eotaxin, levels at 2w during RT of IL-13, IL-15 and eotaxin, and 2w/pre ratio of eotaxin were significantly correlated with grade ≥2 RE (*p* < 0.05). Patients who developed Grade ≥2 RE had lower pre-IL-8 levels than those with grade 0–1 RE (Fig. 1 for comparison of means) (see Fig. 2 for scatter plot of each individual patient).

**Fig. 3:**
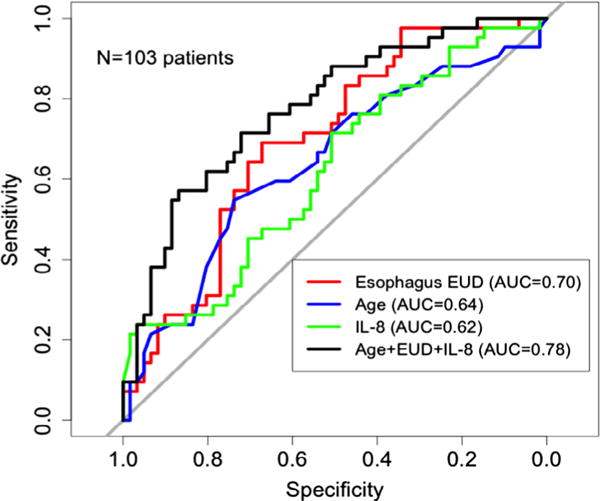
Receiver operating characteristic (ROC) curves of clinical and dosimetric factors (age, esophagus EUD) and cytokines (pre-IL-8) for grade ≥2 RE. RE = radiation esophagitis; EUD = equivalent uniform dose.

Age, esophagus EUD, and baseline IL-8 remained in the final predictive model. The Receiver operating characteristic (ROC) curves showed that the AUC increased to 0.78 by combining IL-8, EUD and age compared with 0.62 by IL-8, 0.64 by age and 0.70 by EUD alone (Fig. 3). The relative predictive ability was significantly higher by combining IL-8, EUD and age than that of EUD and age alone (*p* = 0.019). Cutoff points for age, EUD, and baseline IL-8 were 69 years, 41.4 Gy, and 24.8 pg/ml. Rates of Grade ≥2 RE were 14.2% (5 of 35), 42% (18 of 43), 70% (14 of 20), and 100% (5 of 5) for 0, 1, 2, and 3 risk factors, respectively (*p* < 0.001).

## Discussion

This study demonstrated that age, esophagus EUD, and baseline IL-8 were independent risk factors for Grade ≥2 RE. A model including all three factors performed better than models based on each separately.

Many studies have investigated the predictors of RE, in which grade ≥2 or ≥3 RE was frequently used as the endpoint. The majority of them have focused on dosimetric factors. In our previous study, normal tissue complication probability (NTCP) value was the only significant dosimetric predictor of RE in multivariate analysis [8]. In the QUANTEC study, esophageal volumes receiving >40–50 Gy correlated significantly with acute RE [9]. In current study, esophagus NTCP, EUD, Dmean, Dmax, rV60, aV60, aV70 were all significantly associated with the risk of RE. This study confirmed significance of all the above parameters and demonstrated that EUD remained in the final model to best predict RE with AUC of 0.70 by itself. In the literature, different parameters were identified as the most predictive factors in different studies, including rV20 [10], V35 [11], V50 [12–14], V55 [15], V60 [15], Dmean [16], Dmax [16–18]. We confirmed that esophagus dosimetric parameters were the most important factors for predicting RE, and limiting the irradiation dose to esophagus is the first crucial step to reduce the risk of RE. Since the parameters were well correlated, comprehensive dose-volume histogram evaluation is needed.

Clinical factors, such as younger age [17,19,20], pre-RT dysphagia [17], lower pre-RT body mass index [12], as well as tumor-factors such as nodal stage of N2 or worse [17], have been shown to be correlated with higher risk of RE. Concurrent chemotherapy has been consistently shown to correlate with an increased rate of RE [11,15,16,18,21]. However, in current study, age was the only independent predictive clinical factor, though stage and concurrent chemotherapy were significantly correlated with RE in univariate analysis, as these factors were significantly correlated with each other, age outperformed other factors. Younger age was associated with higher risk of RE, which might be explained that younger patients had increased visceral pain sensation than old ones [22,23].

Though it has been shown that predictive model constructed with dosimetric parameter and clinical factors produced good discriminative ability [24], additional biomarkers help to explain individual variation in patients’ reactions and further improve the predictive ability of RE. In current study, we found IL-8 to be an independent predictor for RE. AUC increased to 0.78 by combining IL-8, EUD and age compared with 0.62 by IL-8, 0.64 by age and 0.70 by EUD alone. IL-8 also increased the predictive ability with addition to EUD and age. Lower baseline IL-8 was associated with higher risk of grade ≥2 RE. To the best knowledge of the authors, this finding has not been reported previously. IL-8 is a cytokine of the CXC chemokine family, playing a role in neutrophil recruitment and activation. Low IL-8 has been reported to be associated with increased risk of radiation-induced lung toxicity in NSCLC [25–27] and the need for percutaneous endoscopic gastrostomy (PEG) tube installation due to mucositis during RT of head and neck squamous cell cancers [28]. However, two studies showed that there was a correlation between RT-induced normal tissue inflammation evaluated by (18)F-fluorodeoxyglucose (FDG) PET and patient outcome. Increased FDG uptake in esophagus after RT was significantly associated with better tumor response and tumor control in esophageal cancer [29], while increased FDG uptake in normal lung and pleura after RT was significantly associated with better tumor response in NSCLC [30], suggesting that tumor radioresponsiveness and normal tissue radiosensitivity may be linked based on shared genetic determinants of inherent radiosensitivity. The immunosuppressive function of IL-8 may lead to decreased side effects but also to worse tumor response. IL-8 as a universal anti-inflammatory cytokine and its role in predicting RT-induced toxicity and tumor response should be further explored.

There are some but not many studies investigating the biomarkers predictive of the risk of RE. The acute phase responses (thrombocytosis and anemia) were found to be associated with RE [31]. Single nucleotide polymorphisms (SNPs) in the TGFβ1 gene were found to be associated with Grade ≥2 or ≥3 RE [2–4] and this finding was validated in one study [3]. High serum miR-155 and miR-221 during the first 2 weeks of concurrent chemoradiotherapy were found to be associated with the development of severe RE [5]. In a model developed by De Ruyck et al., two clinical (nodal stage and gender), four treatment (chemotherapy, Dmean of esophagus, overall treatment time, and RT technique), and four genetic (rs2302535, rs16930129, rs1131877, and rs2230528) parameters were found to be highly predictive for the development of Grade ≥2 RE. The positive predictive value of the biomarker is 69.4%, and the negative predictive value is 87.4% [6]. More works need to be done in finding consistent and clinically useful biomarkers.

Our study has some limitations. Firstly, the sample size and the number of events were not large enough to perform a reliable validation. Secondly, the sample size was also small compared to the high number of tested variables being investigated, which prevented performance of complicated analyses to illustrate the complex interplay between cytokines. Thirdly, there were not enough events to build a model to predict the more clinically important endpoint of RE (Grade ≥3).

In conclusion, age, esophagus EUD, and baseline IL-8 were independent predictors of RE in patients treated with RT for NSCLC. Addition of IL-8 to toxicity model significantly improves RE predictive accuracy. IL-8 as an anti-inflammatory cytokine needs to be validated in large patient populations.

## Figures and Tables

**Table 1:** Baseline patient and treatment related characteristics.

Variables	*N* (%)
*Gender*	
Male	100 (77.5)
Female	29 (22.5)
*Smoking history*	
Never or former smoker	59 (45.8)
Current smoker or recently quit	63 (48.8)
Unknown	7 (5.4)
*Histology*	
Adenocarcinoma	25 (19.4)
Squamous carcinoma	47 (36.4)
Poorly differentiated	42 (32.6)
NOS	15 (11.6)
*Stage*	
I–II	15 (11.6)
III	112 (86.8)
Unknown	2 (1.6)
*Concurrent chemotherapy*	
Yes	88 (68.2)
No	31 (24.0)
Unknown	10 (7.8)
*Radiation dose (Gy)*	
44–59	6 (4.6)
60–69	56 (43.4)
70–79	46 (35.7)
80–85.5	21 (16.3)

**Table 2:** The distribution of dosimetric parameters of esophagus and their association with grade ≥2 radiation esophagitis.

Dosimetric parameter	Median (range)	OR (95% CI)	*p*-Value
Vtotal (cc)	42.7 (22.3-101.5)	0.49 (0.11, 2.24)	0.359
NTCP(%)	11.0 (0-59.7)	1.55 (1.18, 2.03)	0.002
Dmax (Gy)	70.0(2.6-92.2)	22.85 (1.87, 280.00)	0.014
Dmean (Gy)	26.3 (0.3-54.9)	2.72 (1.17, 6.32)	0.020
EUD (Gy)	58.8 (1.4-69.8)	19.74 (1.88, 207.00)	0.013
Veff (%)	3.5 (0-59.8)	1.60 (1.21-2.12)	0.001
rV60 (%)	15.0 (0-52.3)	1.77 (1.24, 2.53)	0.002
aV60 (cc)	6.1 (0-27.7)	1.83 (1.21, 2.77)	0.004
aV70 (cc)	0.0 (0-17.3)	1.92 (1.19, 3.11)	0.008

*Note:* NTCP: normal tissue complication probability, EUD: equivalent uniform dose.

**Table 3:** The association of the levels of 30 cytokines with grade ≥2 RE in 129 NSCLC patients in univariate analysis (cytokine levels are log-transformed).

Cytokine	Absolute pre		Absolute 2w		Absolute 4w		Ratio 2w/pre		Ratio 4w/pre	
	OR(95%CI)	*p*	OR(95%CI)	*p*	OR(95%CI)	*p*	OR(95%CI)	*p*	OR(95%CI)	*p*
EGF	1.01(0.82, 1.23)	0.917	1.05(0.85, 1.29)	0.669	0.93(0.74, 1.17)	0.542	0.99(0.99, 1.00)	0.552	0.99(0.98, 1.01)	0.841
Eotaxin	0.59(0.36, 0.99)	**0.045**	0.51(0.30, 0.86)	**0.012**	0.59(0.34, 1.04)	**0.068**	0.99(0.98, 0.99)	**0.021**	0.99(0.99, 1.00)	0.577
Fractalkine	1.02(0.86, 1.20)	0.820	1.04(0.88, 1.23)	0.627	1.04(0.88, 1.25)	0.636	0.99(0.99, 1.00)	0.193	0.99(0.99, 1.00)	0.119
GCSF	0.88(0.73, 1.05)	0.164	0.82(0.66, 1.02)	0.081	0.94(0.75, 1.17)	0.568	1.00(0.99, 1.00)	0.766	1.00(0.99, 1.00)	0.370
GMCSF	0.97(0.81, 1.16)	0.737	0.95(0.80, 1.14)	0.582	0.96(0.79, 1.16)	0.652	0.99(0.99, 1.00)	0.568	0.99(0.99, 1.00)	0.500
IFN	0.94(0.74, 1.18)	0.578	1.00(0.80, 1.25)	1.000	0.93(0.73, 1.18)	0.566	1.00(0.99, 1.00)	0.580	1.00(0.99, 1.00)	0.634
IL-10	1.08(0.92, 1.27)	0.362	1.04(0.90, 1.21)	0.587	1.12(0.94, 1.33)	0.205	1.00(0.99, 1.01)	0.859	1.00(0.99, 1.01)	0.278
IL-12p40	1.05(0.90, 1.23)	0.503	0.99(0.86, 1.15)	0.929	0.98(0.84, 1.15)	0.837	0.99(0.99, 1.00)	0.386	0.99(0.99, 1.00)	0.473
IL-12p70	1.03(0.86, 1.25)	0.742	0.94(0.77, 1.15)	0.551	0.98(0.80, 1.19)	0.807	0.99(0.98, 1.01)	0.453	1.00(0.99, 1.01)	0.479
IL-13	1.26(1.04, 1.53)	**0.021**	1.24(1.03, 1.51)	**0.026**	1.17(0.96, 1.42)	0.116	0.99(0.99, 1.01)	0.892	0.99(0.98, 1.00)	0.253
IL-15	1.19(0.95, 1.5)	0.124	1.32(1.02, 1.71)	**0.035**	1.30(0.95, 1.78)	0.104	0.99(0.98, 1.01)	0.730	1.00(0.98, 1.02)	0.565
IL-17	1.06(0.88,1.28)	0.517	1.04(0.87, 1.25)	0.681	1.03(0.85, 1.25)	0.763	0.99(0.98, 1.01)	0.627	0.99(0.98, 1.01)	0.588
IL-1	0.95(0.79, 1.14)	0.564	0.97(0.81, 1.15)	0.702	0.89(0.75, 1.06)	0.192	0.99(0.99, 1.00)	0.595	1.00(0.99, 1.00)	0.942
IL-1	1.14(0.96, 1.34)	0.127	1.10(0.93, 1.29)	0.260	1.10(0.94, 1.29)	0.248	0.99(0.98, 1.01)	0.554	0.99(0.97, 1.02)	0.764
IL-1r	1.15(0.98, 1.36)	**0.081**	1.07(0.92, 1.26)	0.376	0.98(0.83, 1.15)	0.776	0.99(0.99, 1.00)	0.221	0.99(0.99, 1.00)	0.204
IL-2	1.15(0.95, 1.39	0.165	1.09(0.91, 1.32)	0.357	1.05(0.87, 1.27)	0.603	0.99(0.98, 1.00)	0.405	1.00(0.99, 1.01)	0.791
IL-4	0.95(0.82, 1.11)	0.535	0.97(0.83, 1.14)	0.721	0.86(0.73, 1.02)	**0.088**	1.00(0.99, 1.01)	0.683	0.99(0.99, 1.00)	0.623
IL-5	1.06(0.89, 1.27)	0.497	1.02(0.86, 1.22)	0.782	0.95(0.81, 1.12)	0.535	0.86(0.72, 1.01)	**0.065**	0.93(0.85, 1.01)	0.083
IL-6	1.02(0.85, 1.22)	0.867	0.95(0.80, 1.15)	0.614	0.93(0.77, 1.12)	0.419	0.99(0.98, 1.01)	0.724	0.99(0.99, 1.01)	0.808
IL-7	1.13(0.89, 1.43)	0.315	0.96(0.76, 1.23)	0.768	1.04(0.81, 1.34)	0.743	0.99(0.96, 1.02)	0.547	0.99(0.97, 1.02)	0.769
IL-8	0.65(0.45, 0.94)	**0.021**	0.73(0.52, 1.01)	**0.054**	0.74(0.52, 1.07)	0.115	0.99(0.98-1.02)	0.717	1.01(0.98, 1.04)	0.331
IP10	0.96(0.77, 1.19)	0.698	0.93(0.75, 1.15)	0.505	0.93(0.75, 1.14)	0.464	1.00(0.99-1.00)	0.690	1.00(0.99-1.10)	0.743
MCP1	1.08(0.75, 1.56)	0.677	1.09(0.81, 1.46)	0.565	0.96(0.68, 1.34)	0.797	0.99(0.99-1.00)	0.344	0.99(0.99, 1.00)	0.527
MIP1	1.03(0.81, 1.3)	0.820	1.00(0.81, 1.23)	0.964	0.96(0.75, 1.24)	0.767	0.99(0.98-1.00)	0.256	0.99(0.99, 1.01)	0.791
MIP1	1.01(0.79, 1.29)	0.923	0.99(0.76, 1.28)	0.942	0.96(0.73, 1.26)	0.791	0.99(0.99-1.00)	0.658	0.99(0.99, 1.00)	0.419
sCD40L	1.05(0.93, 1.19)	0.403	1.05(0.93, 1.19)	0.407	1.02(0.90, 1.15)	0.734	1.00(0.99-1.00)	0.461	1.00(0.99, 1.00)	0.287
TGF	1.21(1.00, 1.47)	**0.054**	1.16(0.97, 1.39)	0.102	1.12(0.93, 1.36)	0.233	1.00(0.97-1.03)	0.891	0.99(0.97, 1.01)	0.434
TNF	0.94(0.67, 1.31)	0.713	1.01(0.77 1.33)	0.945	0.94(0.67, 1.31)	0.716	1.00(0.98-1.02)	0.924	0.99(0.98, 1.02)	0.703
VEGF	0.90(0.73, 1.11)	0.318	0.99(0.79, 1.23)	0.894	0.95(0.77, 1.17)	0.621	0.99(0.99-1.00)	0.590	0.99(0.99, 1.00)	0.241
TGF1	1.20(0.84, 1.71)	0.319	1.11(0.81, 1.53)	0.522	0.93(0.65, 1.32)	0.684	0.95(0.89-1.02)	0.163	0.94(0.87, 1.00)	**0.068**
